# Unknown SARS-CoV-2 pneumonia detected by PET/CT in patients with cancer

**DOI:** 10.1177/0300891620935983

**Published:** 2020-06-22

**Authors:** Maura Scarlattei, Giorgio Baldari, Mario Silva, Stefano Bola, Antonino Sammartano, Silvia Migliari, Tiziano Graziani, Carla Cidda, Nicola Sverzellati, Livia Ruffini

**Affiliations:** 1 Nuclear Medicine Unit, Azienda Ospedaliero-Universitaria di Parma, Parma, Italy; 2 Unit of “Scienze Radiologiche,” Department of Medicine and Surgery, University of Parma, Parma, Italy

**Keywords:** Molecular oncology, diagnostic imaging, thoracic oncology

## Abstract

**Introduction:**

In January 2020, the coronavirus disease 2019 (COVID-19) outbreak in Italy necessitated rigorous application of more restrictive safety procedures in the management and treatment of patients with cancer to ensure patient and staff protection. Identification of respiratory syndrome coronavirus 2 (SARS-CoV-2) infection was a challenge during the pandemic owing to a large number of asymptomatic or mildly symptomatic patients.

**Methods:**

We report 5 patients with unknown SARS-CoV-2 infection undergoing positron emission tomography (PET)/computed tomography (CT) with radiopharmaceuticals targeting different tumor processes: ^18^F-FDG, ^18^F-choline (FCH), and ^68^Ga-PSMA.

**Results:**

In all patients, PET/CT showed increased tracer uptake in the lungs corresponding to CT findings of SARS-CoV-2 pneumonia. Quantitative assessment of tracer uptake showed more elevated values for the glucose analogue ^18^F-FDG (mean SUVmax 5.4) than for the other tracers (mean SUVmax 3.5).

**Conclusions:**

Our findings suggest that PET/CT is a sensitive modality to hypothesize SARS-CoV-2 pneumonia in patients with cancer, even when asymptomatic. More data are needed to verify the correlation among immune response to SARS-CoV-2 infection, clinical evolution, and PET results. Under the strict safety measures implemented at the PET center, the number of potentially SARS-CoV-2–positive patients undergoing PET/CT was very low (1.6%), and no staff member has been diagnosed with infection as of April 30, 2020.

## Introduction

In January 2020, an outbreak of the novel coronavirus disease 2019 (COVID-19) occurred in Italy (https://www.iss.it/en/coronavirus), with striking speed of virus transmission and rapid increase in patient numbers (https://www.ecdc.europa.eu/en/news-events/ecdc-statement-rapid-increase-covid-19-cases-italy). Diagnostic departments were engaged in facing the pandemic with chest radiography and high-resolution computed tomography (HRCT) to assess the presence of pneumonia in patients with respiratory symptoms. In this context, management of diagnostic sessions for patients with cancer required rigorous application of safety procedures with more restrictions compared to routine activity in order to guarantee patient and staff protection.

Identification of patients infected with severe acute respiratory syndrome coronavirus 2 (SARS-CoV-2) appeared early as a challenge of the pandemic, owing to the lack of serologic tests and the absence of testing for infection in the home setting. On the other hand, patients with positive chest computed tomography (CT) findings may have negative reverse transcription polymerase chain reaction (RT-PCR) testing for SARS-CoV-2.^1,2^

Detection of suspected cases is critical in patients with cancer before they visit the positron emission tomography (PET) center, because they are particularly susceptible to respiratory pathogens owing to potential immunosuppressive state and antitumor therapy.

Correct management of a PET session presents many difficulties because of the coexistence of asymptomatic SARS-CoV-2 infection and patients with mild symptoms before the PET scan (https://www.sirm.org/2020/03/30/covid-19-caso-69/), mostly not tested on RT-PCR, but variably presenting with chest CT findings compatible with pulmonary interstitial infiltrates, potentially associated with infection or drug-related reactions.

In this study, we report 5 patients (Table 1) with unknown SARS-CoV-2 infection undergoing PET/CT scan for restaging breast and prostate cancer (patients 1, 3, 4), characterization of lung nodule (patient 2), and focal splenic lesions (patient 5).

**Table 1.** Characteristics of all patients.

Clinical history	**Patient 1**	**Patient 2**	**Patient 3**	**Patient 4**	**Patient 5**
	Solid lung nodule	Breast intraductal cancer	Prostate cancer	Prostate cancer	Splenic lesion
PET indication					
Characterization	Yes	—	—	—	Yes
Staging	—	—	—	Yes	—
Restaging	—	Yes	Yes	—	—
PET tracer	^18^F-FDG	^18^F-FDG	^68^Ga-PSMA	^18^F-choline	^18^F-FDG

PET: positron emission tomography.

## PET/CT imaging protocol

In all cases, PET/CT images were acquired on an integrated 3D PET/CT scanner (Discovery IQ; GE Healthcare, Milwaukee, WI). For PET scanning with ^18^F-FDG, patients fasted for over 6 hours and had blood sugar level <160 mg/dL before intravenous tracer injection.

Whole body PET/CT protocol included a topogram to define the field of view (FOV) established according to the tracer or the cancer type, followed by a low-dose CT scan (120 kV, 140 mA, pitch 1, collimation 16 × 1.25) for attenuation correction and anatomical correlation and a PET emission scan (1.5 minutes per bed position for F-18 tracers, 3 min/bed for Ga-68, 512 × 512 matrix size).

Acquired data were reconstructed by Q Clear (GE Healthcare), a Bayesian penalized-likelihood reconstruction algorithm (strength 350).

Images were corrected for injected dose, tracer decay, body weight, and attenuation using the low-dose CT scan. Informed consent was obtained from all patients.

Review and analysis of attenuation-corrected PET and CT images were performed using an Advantage Workstation 4.6 (GE Healthcare).

Quantitative assessment of tracer uptake was performed by drawing volume of interest (VOI) over areas with abnormally high increases in uptake, focal and/or well-circumscribed.

Standardized uptake value (SUV) was calculated as the ratio of decay-corrected activity in the VOI to the injected activity per unit body weight, as a simplified measure of tracer uptake.

The SUVmax was calculated as the hottest voxel within the VOI.

PET/CT characteristics of all patients are reported in Table 2.

**Table 2.** PET/CT features of all patients.

	Patient 1	Patient 2	Patient 3	Patient 4	Patient 5
Lung involvement	Bilateral	Bilateral	Predominant monolateral	Bilateral	Bilateral
Location	Both central and peripheral	Peripheral	Peripheral	Peripheral	Peripheral
Predominant distribution of opacities	Subpleural	Subpleural	Subpleural	Subpleural	Subpleural
Extent of lesion involvement	Multifocal	Multifocal	Multifocal	Multifocal	Multifocal
Predominant CT pattern	GGO	Mixed pattern (consolidation and GGO)	Mixed pattern (consolidation and GGO)	Mixed pattern (consolidation and GGO)	Mixed pattern (consolidation and GGO)
Lymphadenopathy	Hilar	Mediastinal and hilar	—	Mediastinal	Hilar

CT: computed tomography; GGO: ground-glass opacities; PET: positron emission tomography.

Quantitative analysis of PET images is reported in Tables 3 and 4.

**Table 3.** FDG-PET quantitative assessment of tracer uptake.

	Patient 1	Patient 2	Patient 5
Lung SUVmax	2.6	9.1	4.6
LN SUVmax	2.5	7	2.5

LN: lymph node; PET: positron emission tomography; SUV: standardized uptake value; SUVmax: the hottest voxel within the volume of interest.

**Table 4.** ^68^Ga-PSMA and choline PET quantitative assessment of tracer uptake.

	Patient 3 (^68^Ga-PSMA)	Patient 4 (choline)
Lung SUVmax	3.2	3.8
LN SUVmax	—	3.4

LN: lymph node; PET: positron emission tomography; SUV: standardized uptake value; SUVmax: the hottest voxel within the volume of interest.

## Patient 1 (March 5, 2020)

**Figure 1.**
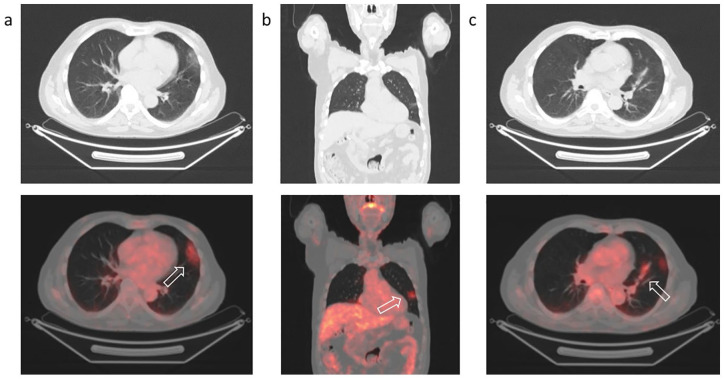
Positron emission tomography/computed tomography with ^18^F-FDG to characterize a solid lung nodule in an 80-year-old man. Increased uptake of ^18^F-FDG (arrows) in subpleural ground-glass opacities of the left lung in the axial (a) and coronal (b) sections with bronchovascular thickening showing mild tracer uptake (c).

A PET/CT scan with ^18^F-FDG (GLUSCAN^®^; AAA, Meldola, FC, IT) was performed to characterize a solid lung nodule (1 cm diameter) revealed by a previous CT scan 1 month previously in an 80-year-old man. The examination was performed 60 minutes after intravenous tracer injection (263 MBq) from scull base to the pelvis. Increased tracer uptake was detected corresponding to subpleural ground-glass opacities (GGOs) on CT images (Figure 1) in the superior segment of the left superior lobe (SUVmax 2.6) with bronchovascular thickening (Figure 1[c]) showing mild tracer uptake (SUVmax 2.5). Active tracer uptake was also present in right hilar lymph nodes (SUVmax 2.5).

The patient, living in a small mountain village, was home-quarantined, but neither swab nor antibodies testing for SARS-CoV-2 was performed. Several weeks later, he underwent lung HRCT, confirming the findings revealed by PET/CT.

## Patient 2 (March 6, 2020)

A 57-year-old woman with a history of intraductal cancer (IDC) was referred for PET for restaging due to appearance of a suspicious nodule in the left breast.

The patient previously (2017) underwent breast-conserving surgery for IDC located in the upper-outer quadrant of the left breast. Follow-up ultrasonography revealed a hypoechoic area (1 cm diameter) near the surgical scar (June 2019); mammography was negative. Fine-needle aspiration of the lesion with ultrasound assistance classified it as benign (C2).

In February 2020, the patient underwent tomosynthesis with evidence of benign alterations (American College of Radiology 2) and absence of microcalcifications or skin alteration suspected of cancer. Ultrasound confirmed the hypoechoic nodule having unchanged size. PET/CT scan with ^18^F-FDG (GLUSCAN^®^) was performed 60 minutes after tracer injection (257 MBq) from vertex to knees for restaging breast cancer.

**Figure 2.**
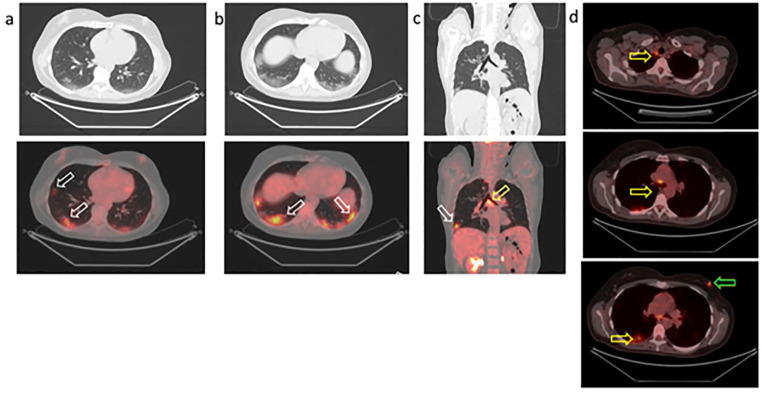
Positron emission tomography/computed tomography with ^18^F-FDG to restage breast cancer in a 57-year-old woman. Increased uptake of ^18^F-FDG (white arrows) in subpleural ground-glass opacities of both lungs in the axial (a, b) and coronal (c) sections. Tracer uptake in the carinal (c) and mediastinal (d) lymph nodes due to infection (yellow arrows). Focal tracer uptake in the left breast corresponds to the suspected nodule (d; green arrow). Note: The colour version of the figure is available online.

Focal tracer uptake (SUVmax 6.5) was detected in the solid nodule of the left breast. Intense and diffuse uptake was revealed in both lungs corresponding to GGOs on CT images (Figure 2; also see the maximum intensity projection in Supplementary Figure 1).

CT characteristics were typical of COVID-19 pneumonia as described by Xie et al.^1^ Tracer uptake was present in all parenchymal alterations with different intensity (SUVmax range in the right lung, 2.2–7.7; SUVmax range in the left lung, 2.7–9.1).

The highest SUV values were measured in the subpleural GGO areas; otherwise, low uptake values were revealed in the isolated lesions in the parenchyma. High intensity of tracer uptake present in mediastinal, hilar, and carinal lymph nodes refer to infection (SUVmax 7).

The patient was referred to the dedicated COVID-19 protocol at the University Hospital of Parma and subsequently home-quarantined because she did not show any symptoms. Her general practitioner was alerted for peripheral management of the patient and organization of pharyngeal swab for testing for SARS-CoV-2 infection. The swab was not performed, but the patient underwent a rapid diagnostic test for SARS-CoV-2 antibodies from a finger prick, which resulted positive for immunoglobulin G (IgG).

## Patient 3 (March 24, 2020)

A 65-year-old man had previously undergone radical prostatectomy for adenocarcinoma Gleason Score 7 (4+3). During follow-up, his prostate-specific antigen (PSA) value slowly increased, with an actual value of 0.47 ng/mL, leading to a need to restage prostate cancer with ^68^Ga-PSMA PET/CT.

**Figure 3.**
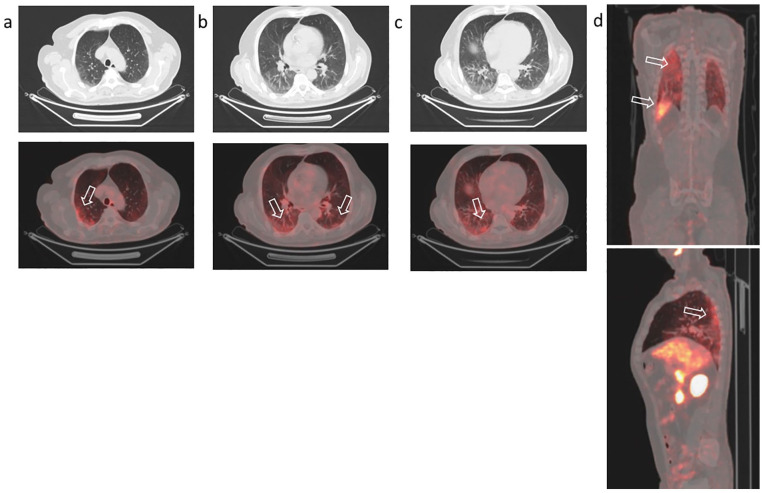
Positron emission tomography/computed tomography with ^68^Ga-PSMA to restage prostate cancer (Gleason Score 7, prostate-specific antigen 0.4 ng/mL) in a 65-year-old man who previously had radical prostatectomy. Increased uptake of ^68^Ga-PSMA in areas of ground-glass opacities (arrows) in the posterior segments of both lungs. Axial coronal (a, b, c) and sagittal (d) sections are shown.

The radiopharmaceutical was prepared in our radiopharmacy laboratory as described previously^3^ according to current European Union Good Manufacturing Practices,^4^ current Good Radiopharmacy Practice,^5^ and European Pharmacopoeia. Dynamic images over the pelvis were acquired soon after intravenous tracer injection (142 MBq). Whole-body scan was performed after an uptake time of 60 minutes from vertex to knee. PET/CT was negative for cancer lesions but revealed mild tracer uptake (SUVmax 3.2) in the subpleural region of both lungs, with greater extent in the right lung, corresponding to CT findings of subpleural GGOs in the dependent lung (Figure 3). Subsequently, the patient underwent pharyngeal swab testing, which was positive. After symptoms disappearance, another swab was performed, resulting negative. Several weeks later, the patient’s wife presented symptoms of SARS-CoV-2 infection (cough, fever, diffuse myalgia), treated with hydroxychloroquine and azithromycin, not requiring hospitalization. She is awaiting serologic testing.

## Patient 4 (March 25, 2020)

A 70-year-old man previously underwent standard prostate mapping due to PSA value increase (last value 7.4 ng/mL). Adenocarcinoma of the prostate was demonstrated in 9/12 mapping samples with a Gleason Score 7 (3+4).

A PET/CT scan with ^18^F-choline (IASOcholine^®^; IASON GmbH, Graz, Austria) was performed for staging. Dynamic images of the pelvis were acquired soon after intravenous tracer injection (278 MBq). The whole-body scan was performed 60 minutes after intravenous tracer injection from scull base to mid-thighs.

**Figure 4.**
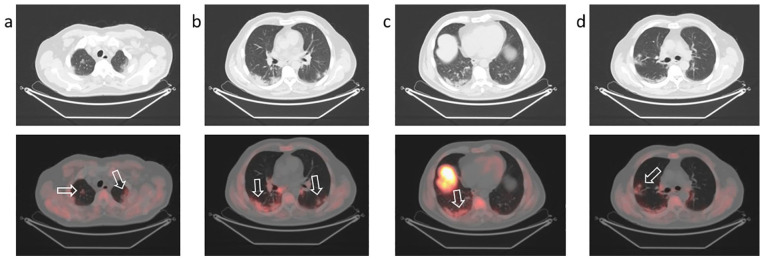
Positron emission tomography/computed tomography with ^18^F-choline to stage prostate cancer (Gleason Score 7, prostate-specific antigen 7.4 ng/mL) in a 70-year-old man. Increased uptake of ^18^F-choline (arrows) in peripheral ground-glass and consolidative opacities (a, b, c, d) in both lungs (major extent in the right lower lobe).

PET/CT showed double focal intense uptake in the prostate gland. Mild uptake of the tracer was revealed in the subpleural region of both lungs, with greater extent in the right lung, corresponding to CT findings of GGOs and consolidation areas (Figure 4). Focal tracer uptake (SUVmax 3.8) was detected in a single GGO localized in the middle lobe of the right lung and in a hilar, carinal, and peribronchial lymph node (SUVmax 3.4) (see Supplementary Figure 2).

The patient’s general practitioner was alerted for peripheral management and organization of pharyngeal swab testing for SARS-CoV-2 infection. The patient was home-quarantined but swab was not performed by decision of the Health Department. He recently underwent laboratory-based serologic testing, which was positive for IgG antibodies to SARS-CoV-2.

## Patient 5 (March 26, 2020)

A 57-year-old woman underwent PET/CT with ^18^F-FDG (GLUSCAN^®^) to characterize three focal splenic lesions (diameter 8–10 mm) with inconclusive CT and contrast-enhanced ultrasound. The examination was performed 60 minutes after intravenous tracer injection from skull base to pelvis. PET/CT did not show any tracer uptake in the spleen but revealed intense/moderate uptake in the bilateral subpleural regions (SUVmax 4.6 in the right lung, SUVmax 3.7 in the left lung) corresponding to CT findings of GGOs in the posterior segments, with obvious extent in the right lung (see Supplementary Figure 3).

The patient developed mild respiratory symptoms a few weeks later; she was quarantined and recently underwent thoracic radiography, which was normal.

## Discussion and conclusion

To our knowledge, this is the first report of SARS-CoV-2 infection in patients with cancer detected by PET/CT using different tracers according to tumor type.

In a 1-month period (March 2020), we performed 302 PET/CT examinations for patients with cancer. Five patients (1.6 %) undergoing PET/CT revealed PET manifestations corresponding to CT findings suspicious for SARS-CoV-2 infection. All of them were asymptomatic on the day of PET/CT scan; one of them (case 4) questioned after the examination reported a mild fever 3 weeks previously.

**Figure 5.**
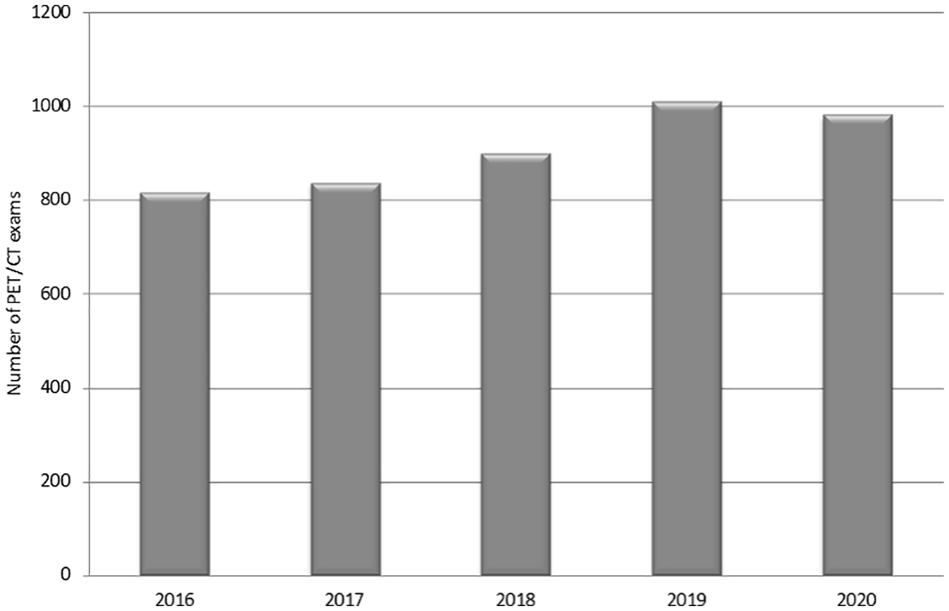
Total positron emission tomography (PET)/computed tomography (CT) examinations in the first trimester (years 2016–2020) at the PET Center of the University Hospital of Parma.

**Figure 6.**
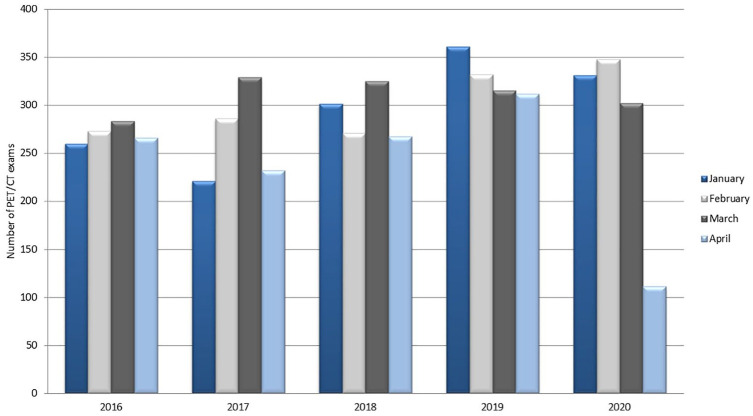
Positron emission tomography (PET)/computed tomography (CT) examinations per month for patients with cancer (data collected until April 30 for 2020).

In Figures 5 and 6, activity of the PET center during the outbreak is reported compared to previous years (for 2020, activity was measured until April 30).

Patient 3 was from Codogno, the first red zone area for SARS-CoV-2 infection in Italy (https://www.escardio.org/Education/COVID-19-and-Cardiology/diagnosing-the-first-covid-19-patient-in-italy-codogno). He presented symptoms (ageusia, cough) immediately after the identification of the man known in Italy as Patient No. 1.^6^ After disappearance of symptoms, he underwent pharyngeal swab testing for SARS-CoV-2 infection, which resulted negative. A second swab was not performed. Some weeks later, the patient’s wife presented symptoms of SARS-CoV-2 infection. She is now awaiting serologic testing. Most patients (patients 1, 2, 4, 5) home-quarantined after PET scan, awaiting swab testing, but it was performed only in one case (patient 3), due to the lack of symptoms. However, recent implementation of serologic testing has allowed confirmation of previous SARS-CoV-2 infection in two cases (patients 2 and 4).

Our findings suggest that PET/CT is a sensitive modality to demonstrate the presence of SARS-CoV-2 pneumonia in patients with cancer, even in asymptomatic individuals.

Persistence of tracer uptake many weeks after symptoms disappearance (patient 3) may be explained by the presence of inflammatory cells accumulating ^18^F-FDG (neutrophils, activated macrophages, and lymphocytes) at the site of infection, without excluding active disease. In inflamed tissue, glycolysis is enhanced^7^ with an upregulation of GLUT transporters, with amplified affinity for their substrates as glucose analogue ^18^F-FDG.

Specific infections, such as human immunodeficiency virus, cytomegalovirus, or dengue virus, may lead to increased expression of GLUT transporters in infected cells,^8–10^ with a parallel rise in FDG accumulation.^8–10^ However, in our patients, tracers targeting different metabolic process, such as membrane biosynthesis (^18^F-choline) or the overexpression of transmembrane glycoprotein (^68^Ga-PSMA), showed moderate to intense uptake in the typical COVID-19 pneumonia.

Increased regional blood flow at the site of infection/inflammation may increase availability of PSMA ligand. Additionally, folate receptors overexpressed by activated macrophages may interfere with the expression of folate hydrolase/PSMA.^11^ FCH accumulation in inflammatory tissue may be due to upregulation of choline kinase in the activated macrophages.^12^

More data are needed to verify the correlation among immune response to SARS-CoV-2 infection, clinical evolution, and PET results; patient 3 had negative swab after symptom disappearance but positive PET examination for bilateral subpleural GGOs, and his wife later developed symptoms of COVID-19.

In our cases, different types of serologic testing were used, such as rapid diagnostic test in patient 2 and laboratory-based test in patient 4, allowing us to establish a previous SARS-CoV-2 infection. More homogeneous management of pharyngeal swab and serologic testing could help infection tracing, especially in the vulnerable population of patients with cancer with PET/CT findings suspicious for SARS-CoV-2 pneumonia.

Recent case series about PET in patients with SARS-CoV-2 pneumonia showed high ^18^F-FDG uptake in lung lesions accompanied by nodal involvement detectable on PET/CT images.^13^ Incidental findings of SARS-CoV-2 infection in asymptomatic patients with cancer undergoing nuclear medicine procedures was recently reported using hybrid scanner single-photon emission CT/CT^14^ and PET/CT.^14,15^

The PET tracer used was the glucose analogue ^18^F-FDG in all the reported cases. We have shown that different radiopharmaceuticals, used according to tumor type, may be taken up by SARS-CoV-2 pneumonia. In our cases, quantitative assessment of ^18^F-FDG uptake was higher in the parenchymal lesions than in the accompanying nodal involvement. The SUVmax values of ^18^F-FDG were more elevated compared to the other tracers (mean value 5.4 vs 3.5) assessed previously in preclinical experiments.^12^

These preliminary data show prolonged metabolic activity in the typical lung lesions of SARS-CoV-2 infection, remaining many weeks after symptoms disappearance or negative swab (patient 3), suggesting a potential role of PET/CT in monitoring treatment efficacy and disease resolution. We hypothesize that quantitative results of tracer uptake measured by SUV may be referred to infection timing, with higher SUVs in the initial phase of pulmonary involvement (patient 2).

Under the strict protective measures implemented at the PET Center, the number of potentially SARS-CoV-2–positive patients undergoing PET/CT was very low, and no staff member has been diagnosed with SARS-CoV-2 infection as of April 30, 2020, as confirmed by negative results of serology testing (immunoglobulin M and IgG) for SARS-CoV-2, performed at the beginning of May, in all staff members.

## Supplemental Material

Supplemental_figures_1 – Supplemental material for Unknown SARS-CoV-2 pneumonia detected by PET/CT in patients with cancer Click here for additional data file. Supplemental material, Supplemental_figures_1 for Unknown SARS-CoV-2 pneumonia detected by PET/CT in patients with cancer by Maura Scarlattei, Giorgio Baldari, Mario Silva, Stefano Bola, Antonino Sammartano, Silvia Migliari, Tiziano Graziani, Carla Cidda, Nicola Sverzellati and Livia Ruffini in Tumori Journal

Supplemental_figures_2 – Supplemental material for Unknown SARS-CoV-2 pneumonia detected by PET/CT in patients with cancer Click here for additional data file. Supplemental material, Supplemental_figures_2 for Unknown SARS-CoV-2 pneumonia detected by PET/CT in patients with cancer by Maura Scarlattei, Giorgio Baldari, Mario Silva, Stefano Bola, Antonino Sammartano, Silvia Migliari, Tiziano Graziani, Carla Cidda, Nicola Sverzellati and Livia Ruffini in Tumori Journal

Supplemental_figures_3 – Supplemental material for Unknown SARS-CoV-2 pneumonia detected by PET/CT in patients with cancer Click here for additional data file. Supplemental material, Supplemental_figures_3 for Unknown SARS-CoV-2 pneumonia detected by PET/CT in patients with cancer by Maura Scarlattei, Giorgio Baldari, Mario Silva, Stefano Bola, Antonino Sammartano, Silvia Migliari, Tiziano Graziani, Carla Cidda, Nicola Sverzellati and Livia Ruffini in Tumori Journal
