# Supplementary figures and images for: Unknown SARS-CoV-2 pneumonia detected by PET/CT in patients with cancer
Source: Tumori. 2020 Jun 22;106(4):325–32. doi: 10.1177/0300891620935983 (PMC7308791; doi:10.1177/0300891620935983)

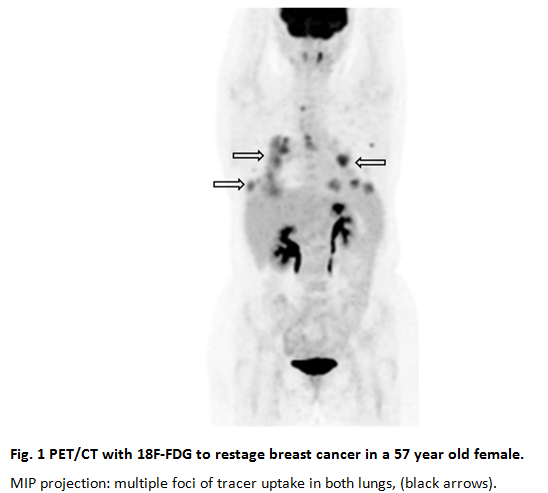

Supplement: Supplemental_figures_1 – Supplemental material for Unknown SARS-CoV-2 pneumonia detected by PET/CT in patients with cancer [file Supplemental_figures_1.tiff]

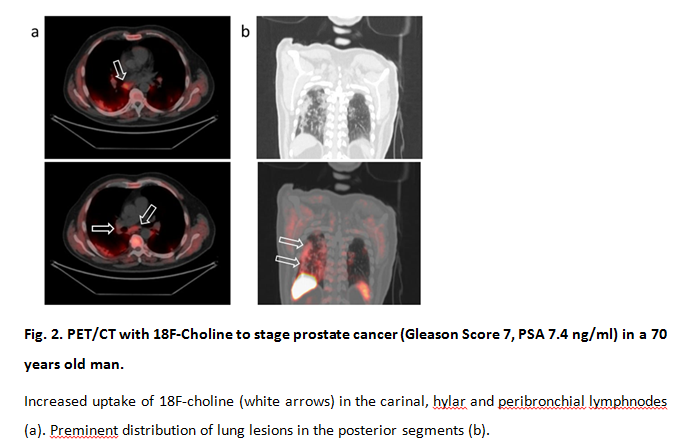

Supplement: Supplemental_figures_2 – Supplemental material for Unknown SARS-CoV-2 pneumonia detected by PET/CT in patients with cancer [file Supplemental_figures_2.tiff]

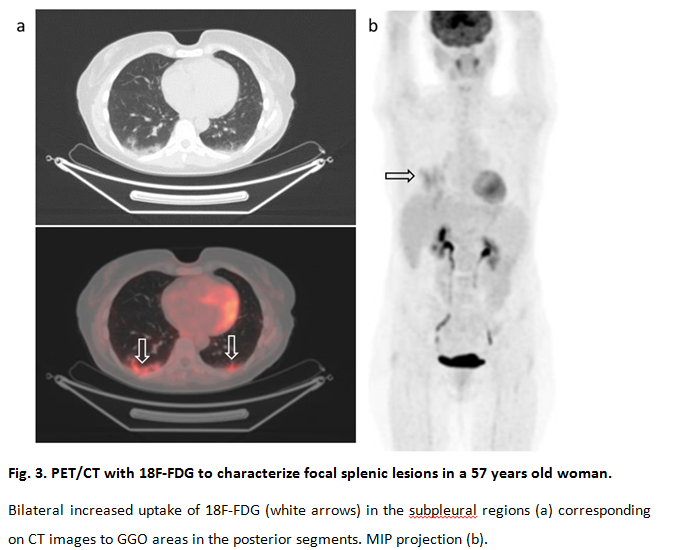

Supplement: Supplemental_figures_3 – Supplemental material for Unknown SARS-CoV-2 pneumonia detected by PET/CT in patients with cancer [file Supplemental_figures_3.tiff]
